# Sustainable Alginate–Hydrochar Composite Beads for 2-Nitrophenol Adsorption in Batch and Fixed-Bed Systems

**DOI:** 10.3390/ma18102412

**Published:** 2025-05-21

**Authors:** Dalia Allouss, Nicolas Abatzoglou, Inès Esma Achouri

**Affiliations:** 1 Group of Research on Technologies and Processes, Department of Chemical and Biotechnological Engineering, Université de Sherbrooke, Sherbrooke, QC J1K 2R1, Canada; dalia.allouss@usherbrooke.ca (D.A.); nicolas.abatzoglou@usherbrooke.ca (N.A.); 2 Canada Research Chair on Process Intensification for Advanced Catalyst and Sustainable Energy, Université de Sherbrooke, Sherbrooke, QC J1K 2R1, Canada

**Keywords:** 2-Nitrophenol, hydrochar, immobilization, adsorption, fixed-bed, modeling, wastewater treatment

## Abstract

Addressing the removal of hazardous phenolic pollutants from water, this study introduces an eco-friendly adsorbent composed of waste-derived hydrochar immobilized in alginate beads (Alg/HC). The physicochemical properties of the Alg/HC beads were characterized using SEM, XRD, and FTIR, confirming hydrochar encapsulation and partial structural preservation. Batch studies revealed a maximum 2-nitrophenol (2-NP) adsorption capacity of 15.80 ± 0.62 mg/g at 30 mg/L of 2-NP, with kinetics best described by the Elovich and pseudo-second-order models. Freundlich isotherm fitting indicated multilayer adsorption on heterogeneous surfaces, likely governed by hydrogen bonding and π–π interactions. In a fixed-bed column system, Alg/HC beads demonstrated a continuous adsorption capacity of 6.84 ± 0.45 mg/g at 10 mg/L of 2-NP, with breakthrough behavior modeled by the Yoon–Nelson and Thomas equations. The beads maintained stable performance across four regeneration cycles using a mild water/ethanol desorption method. This work represents the first study to explore Alg/HC composites for 2-NP removal in both batch and continuous modes, demonstrating their potential as low-cost, regenerable adsorbents for tertiary treatment of phenolic industrial wastewater.

## 1. Introduction

The pollution of water sources with emerging pollutants—including pharmaceuticals, pesticides, heavy metals, and phenolic compounds—poses serious ecological and public health risks in Canada and globally [1,2,3]. Among these, nitrophenols are of primary focus due to their toxicity, persistence, and resistance to biodegradation. Specifically, 2-nitrophenol (2-NP), classified as a priority pollutant by the U.S. EPA, frequently enters aquatic systems through industrial discharges, even at trace concentrations [4,5].

Various treatment technologies have been investigated for 2-NP removal, including membrane filtration, photocatalysis, electrochemical methods, and adsorption [6,7,8,9]. Among these, adsorption is a cost-effective and efficient method due to its simplicity and adaptability to diverse water matrices [10,11]. However, most reported adsorbents are powdered materials that suffer from key drawbacks such as difficult post-treatment recovery and poor suitability for continuous systems due to clogging risks [12]. This presents a key limitation for scaling adsorption technologies to industrial or municipal wastewater treatment.

Hydrothermal carbonization offers a sustainable approach to valorizing waste biomass into hydrochar (HC), a carbon-rich material with promising adsorption properties [9,13,14]. Yet, its powdered form limits application in fixed-bed systems. To address this challenge, this study introduces a novel, immobilized composite system based on alginate–hydrochar (Alg/HC) beads, synthesized via ionotropic gelation and suitable for both batch and continuous operations. This design improves material manipulation, structural strength, and environmental potential through surface chemistry, without the need for chemical or thermal activation. Recent advances in adsorbent design have focused on immobilizing functional materials within biopolymeric matrices to improve mechanical strength, reusability, and flow dynamics. Among these, alginate is particularly attractive due to its renewability, biocompatibility, and mild gelation properties, making it an ideal carrier for forming composite hydrogel beads [15,16].

Although hydrochar-based adsorbents have been widely studied for organic pollutant removal in batch systems, their application in fixed-bed column systems, particularly for nitrophenol removal, is still underexplored. Furthermore, studies evaluating adsorbents at low, environmentally relevant pollutant concentrations (e.g., 10 mg/L) and in continuous flow conditions remain relatively limited. To our knowledge, this is the first study to investigate Alg/HC beads for the removal of 2-NP in batch and continuous systems, providing a preview of real-world applications.

The beads were characterized using SEM, FTIR, and XRD, and tested for 2-NP removal in batch and fixed-bed column modes. Kinetic and isotherm models were applied to elucidate adsorption mechanisms, while breakthrough modeling was used to predict column performance. Finally, reusability was assessed over multiple adsorption–regeneration cycles to assess the viability of Alg/HC as a scalable, eco-friendly adsorbent for tertiary wastewater treatment.

## 2. Materials and Methods

### 2.1. Materials

All chemicals used were of analytical grade and used without further purification. Solutions were prepared using distilled water (DW). Sodium alginate (Alg) and 2-NP (98% purity, chemical formula O_2_NC_6_H_4_OH; molar mass 139.11 g/mol, CAS no. 88–75-5) were purchased from Sigma Aldrich (St. Louis, MO, USA). The HC, derived from Cu-laden switchgrass biosorption waste provided by the Centre de recherche sur les grains (CÉROM, QC, Canada), was synthesized via hydrothermal carbonization as reported in our previous study [9]. A fresh stock solution of 2-NP (500 mg/L) was prepared prior to each experiment to ensure stability.

### 2.2. Alginate–Hydrochar Composite Bead Preparation

Alginate–hydrochar (Alg/HC) composite beads were prepared using the ionotropic gelation technique. A homogeneous suspension was obtained by dispersing 0.5 g of HC in 100 mL of DW, followed by 10 min of sonication. Then, 3 g of Alg was gently added under continuous stirring (500 rpm) at room temperature, and the mixture was stirred overnight to secure uniform gel formation. The Alg/HC hydrogel beads were prepared using the ionotropic gelation method [17]. The Alg/HC mixture was added dropwise into a 4% *w*/*v* CaCl_2_ dihydrate solution in a crystallizing dish using a peristaltic pump (7518-60 Masterflex L/S) under gentle agitation and was left overnight to mature at ambient temperature. The experimental setup is illustrated in the supplementary data (Figure S1). The beads were then separated from the coagulation bath, thoroughly washed using DW to remove excess Ca^2+^ ions, and lyophilized for further characterization. The Alg/HC hydrogel beads were spherical, with an average diameter between 2.5 and 2.7 mm (Figure S2). The hydrogel beads were stored in glass vials filled with DW for future applications.

### 2.3. Characterization

The presence of functional groups and changes in surface chemical bonding were studied using FTIR spectroscopy. Spectra were recorded over the 500–4000 cm^−1^ wavenumber range using an Agilent Cary 630 instrument equipped with an attenuated total reflection (ATR) system. The crystallinity of the Alg/HC beads was determined through XRD analysis using an X’Pert Pro MPD (Panalytical, Malvern, UK) with Cu Kα radiation (λ = 1.54 Å) over a 2θ range of 10–80°. The morphology and texture of the Alg/HC beads were examined using a Thermo Fisher Scientific Phenom XL G1 SEM (Waltham, MA, USA). To evaluate the copper leaching potential from the Alg/HC hydrogel beads, inductively coupled plasma (ICP) spectroscopy (PerkinElmer Avio 500) was used. The test was conducted in duplicate by analyzing the filtrate from batch adsorption assays using ICP to measure Cu concentration after 1 h of contact time.

### 2.4. 2-Nitrophenol Batch Adsorption Experiments

A series of experiments was conducted in 50 mL beakers, each containing 25 mL of 2-NP solution. A measured quantity of the Alg/HC hydrogel beads was added to the solutions. The influence of contact time (10–180 min), initial 2-NP concentration (2–30 mg/L), sorbent bead dose (0.4–2 g/L), and adsorption temperature (25, 35, and 45 °C) was investigated under unadjusted pH using an orbital shaker at 200 rpm—to determine the adsorption kinetics, isotherms, and thermodynamics in the batch studies. All the adsorption experiments were conducted in duplicate, with a control experiment (without HC in the hydrogel beads). After the experiments, the residual 2-NP concentration was measured using an ultraviolet–visible (UV-Vis) spectrophotometer (Ultrospec 2100 Pro) at 350 nm, determined via a calibration curve. The adsorption capacity (*q_e_,* mg/g) and the removal efficiency (*R*%) of the Alg/HC beads were calculated using standard adsorption equations, which are provided in the supplementary data. Adsorption parameters were determined based on dry weight, with 43 mg of lyophilized beads equivalent to 1 g of hydrogel beads. The details of the fitting formulas for the adsorption kinetics, isotherm, and thermodynamics models are provided in the supplementary data.

### 2.5. Continuous Column Adsorption Experiments

**Figure 1:**
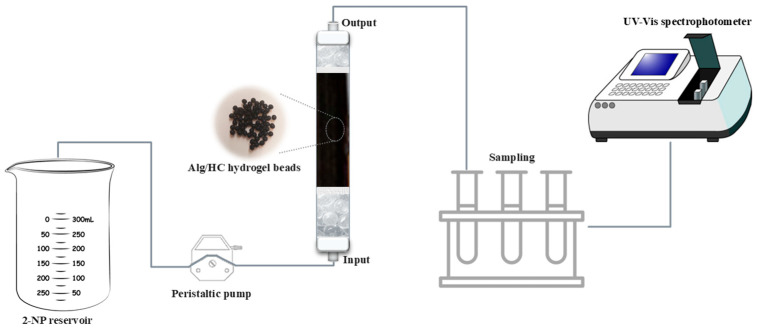
Representation of the continuous-flow fixed-bed column adsorption experiment.

The column experiments were conducted using a laboratory-scale plexiglass column with an inner diameter of 2 cm and a length of 60 cm. The column was positioned vertically, and the 2-NP solution was continuously introduced through the inlet at the bottom of the reactor in upflow mode using a peristaltic pump. This configuration prevented air bubble entry, which could cause channeling within the column. Support layers of quartz wool and glass beads were placed at the column inlet and outlet to evenly distribute the Alg/HC hydrogel beads and prevent compaction or channeling. The effects of key process variables—inflow rate, initial 2-NP concentration, and bed height—were investigated by analyzing breakthrough curves. All the tests were conducted at room temperature (25 ± 2 °C). The continuous adsorption experimental setup is illustrated in Figure 1.

#### Determination of the Column Parameters

Breakthrough curves for the continuous 2-NP adsorption were plotted using concentration ratio (*C*_t_/*C*_0_) versus time (*t*) under various operating conditions. The breakthrough time (*t*_b_) is the point when the effluent concentration reaches approximately 10% of the initial effluent concentration [18]. The saturation time (*t*_s_) is the point at which the 2-NP effluent concentration exceeds 90% of the influent concentration, indicating that the adsorbent is fully exhausted. The change in the residual concentrations in the effluent over time enabled the calculation of the total amount adsorbed (*q*_total_) (mg) using the following equation [19,20]:

(1) $$q_{\mathrm{total}}\;=\frac{Q}{1000}\int_{t=0}^{t=t_{\mathrm{total}}}(C_{0}-C_{t})\;dt$$

where *Q* is the flow rate (mL/min), *C*_0_ and *C*_t_ are the inlet and outlet concentrations of 2-NP (mg/L), and *t*_total_ is the total flow time elapsed to reach column saturation.

The adsorption capacity (*q*_e_) (mg/g) of the column was calculated using the following equation:

(2) $$q_{e}=\frac{q_{\mathrm{total}}}{m}$$

where *m* is the dry weight of the Alg/HC wet beads in the fixed-bed column (g).

The total amount of 2-NP added to the column (*m*_total_) (mg) was calculated as follows:

(3) $$m_{\mathrm{total}}=\frac{C_{0}Qt_{\mathrm{total}}}{1000}$$

The removal percentage of 2-NP for the fixed-bed column was calculated as follows:

(4) $$R\left(\%\right)=\frac{q_{\mathrm{total}}}{m_{\mathrm{total}}}\times 100$$

The effluent volume (*V*_eff_) was calculated as follows:

(5) $$V_{\mathrm{eff}}=Qt_{\mathrm{total}}$$

The length of the mass transfer area (*Z*_m_) and the adsorption zone (Δ*t*) were determined using the breakthrough curves and calculated using the following equations [21]:

(6) $$Z_{m}=Z(1-\frac{t_{b}}{t_{e}})$$

(7) $$\Delta t=t_{s}-t_{b}$$

### 2.6. Regeneration Study in the Continuous System

In this study, adsorption–desorption experiments were conducted over four consecutive cycles to assess the regeneration potential of the Alg/HC hydrogel beads in a continuous packed-bed system operated at ambient temperature. The beads were initially loaded with 2-NP at a concentration of 10 mg/L, using a flow rate of 5.2 mL/min and a bed height of 15 cm. Upon saturation, the remaining 2-NP solution was drained from the column before regeneration. The adsorbent was regenerated using a 1:1 (*v*/*v*) DW/ethanol solution, introduced in upflow mode. After desorption, the regenerated adsorbent bed was rinsed with DW several times before the next adsorption cycle. Adsorption and desorption capacities were systematically evaluated after each cycle to assess bead performance over the four cycles.

### 2.7. Goodness-of-Fit Analysis

The fit between adsorption models and experimental data was assessed using the coefficient of determination (R^2^), the adjusted coefficient of determination (R_Adj_^2^), the root means square error (RMSE), and the corrected Akaike information criterion (AIC_c_) [20,22]. AIC_c_ is widely used for model selection and ranking in various research fields, including adsorption studies [23]. Model selection was based on minimizing AIC_c_ and RMSE and maximizing R^2^ and R_Adj_^2^.

## 3. Results and Discussion

### 3.1. Alginate–Hydrochar Adsorbent Characterization

**Figure 2:**
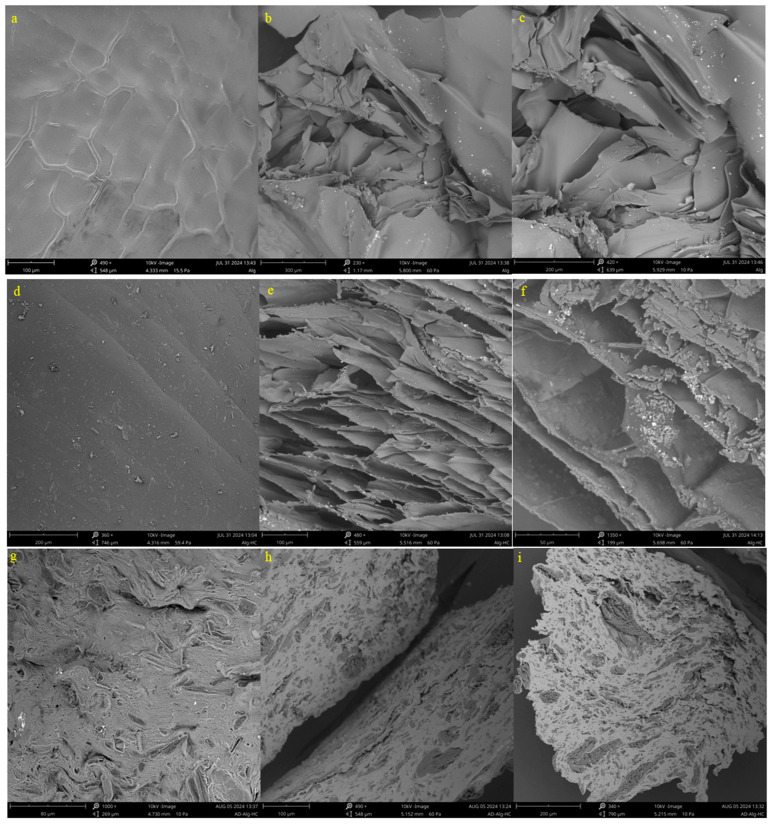
SEM images of the lyophilized (**a**) Alg bead and (**d**) Alg/HC bead and the air-dried (**g**) Alg/HC bead; cross-sections of the lyophilized (**b**,**c**) Alg bead and (**e**,**f**) Alg/HC bead and the air-dried (**h**,**i**) Alg/HC bead.

The synthesized Alg/HC beads were characterized using scanning electron microscopy (SEM), X-ray diffraction (XRD), and Fourier transform infrared (FTIR) spectroscopy to assess morphology, crystallinity, and chemical interactions. SEM images of both surface and cross-sectional morphologies of the lyophilized and air-dried beads are presented in Figure 2. The freeze-dried Alg bead surface had a smooth texture with minor rippling (Figure 2a), whereas the lyophilized Alg/HC beads showed a denser, more uniform surface with immobilized HC, indicating effective integration into the polymeric matrix (Figure 2d). In contrast, the air-dried Alg/HC beads exhibited a rough surface with noticeable cavities (Figure 2g), attributed to hydrogel matrix collapse during air-drying, which likely induced structural defects [24,25]. Cross-sectional SEM images revealed structural differences. The lyophilized Alg beads had a folded internal morphology with visible cracks (Figure 2b,c), while the lyophilized Alg/HC beads exhibited densely packed, layered structures with dark embedded particles corresponding to copper-rich hydrochar (Figure 2e,f) [9]. The air-dried Alg/HC beads displayed a distinctive brain-like texture with dispersed dark regions, confirming successful HC immobilization and enhanced internal porosity (Figure 2e–i).

**Figure 3:**
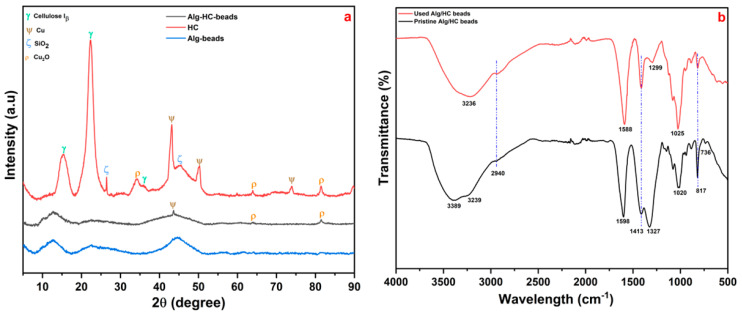
(**a**) XRD and (**b**) FTIR spectra of the hydrogel bead adsorbent.

The XRD pattern of the unfilled calcium alginate beads is shown in Figure 3a. The unfilled Alg beads exhibited two characteristic crystalline peaks at 2θ = 12.63° and 22.46°, corresponding to the semi-crystalline nature of the calcium crosslinked alginate matrix [26,27]. The HC exhibited broad cellulose-associated peaks at 15.42° and 22.38°, attributed to residual biomass lattice structures [9,28]. In comparison, the XRD pattern of Alg/HC beads showed reduced peak intensities and slight shifts in the crystalline peaks of HC, suggesting structural modifications and successful HC incorporation into the Alg matrix.

FTIR spectroscopy provided additional evidence of functional group interactions (Figure 3b). The pristine Alg/HC beads showed broad peaks between 3389 and 3239 cm^−1^, characteristic of hydroxyl groups (O–H), suggesting hydrogen bonding between the alginate and hydrochar materials [9,29]. The absorption band at 2940 cm^−1^ was assigned to C–H stretching. The asymmetric and symmetric stretching vibrations of carboxyl (COOH) groups appeared at 1598 cm^−1^ and 1413 cm^−1^, respectively. In the used Alg/HC beads, the COOH asymmetric stretching peak shifted from 1598 cm^−1^ to 1588 cm^−1^, indicating chemical interactions between carboxyl groups and adsorbed 2-NP molecules. Similarly, the skeletal vibration peak of Alg at 1327 cm^−1^ (C–OH stretching) shifted to 1299 cm^−1^, suggesting structural changes in the Alg matrix. Bands at 817 cm^−1^ and 736 cm^−1^ were assigned to mannuronic and guluronic acids, confirming the presence of Alg components [27,30]. Collectively, these observations indicate that hydroxyl, alkoxy, and carboxyl functional groups participated in 2-NP binding. The observed spectral shifts support a dominant adsorption mechanism involving hydrogen bonding, electrostatic attraction, and π–π interactions [13,31].

### 3.2. Batch Adsorption Performance

**Figure 4:**
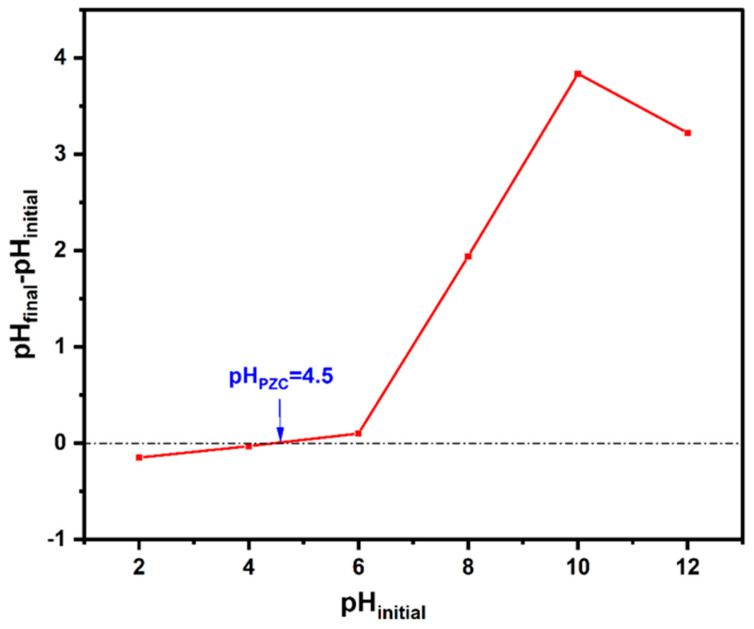
Point zero charge of the Alg/HC hydrogel beads.

Batch adsorption experiments were conducted using 2-nitrophenol (2-NP) solutions at an initial concentration of 10 mg/L to compare the performance of lyophilized and wet alginate (Alg) and alginate–hydrochar (Alg/HC) beads. Two adsorbent doses were tested: 0.68 g/L and 2 g/L. All experiments were performed at pH 5.2, corresponding to the natural pH of the prepared 2-NP solution. The pK_a_ of 2-NP is 7.17 at 25 °C [32], while the point of zero charge (pH_PZC_) of the Alg/HC beads was determined to be 4.5 (Figure 4). At pH 5.2—below the pKa—2-NP predominantly exists in its neutral molecular form, which facilitates hydrogen bonding with the negatively charged adsorbent surface. Additionally, 2-NP is an electron-rich aromatic compound with asymmetric charge distribution: the nitro group (NO₂) carries partial negative charge, while the delocalized aromatic ring contributes to polar interactions. This behavior enhances adsorption potential through π–π interactions and *n*→π* interactions between aromatic rings of 2-NP and adsorbent surface. The selected pH was consistent with previous studies that report optimal 2-NP adsorption near pH 5.5 [31].

**Figure 5:**
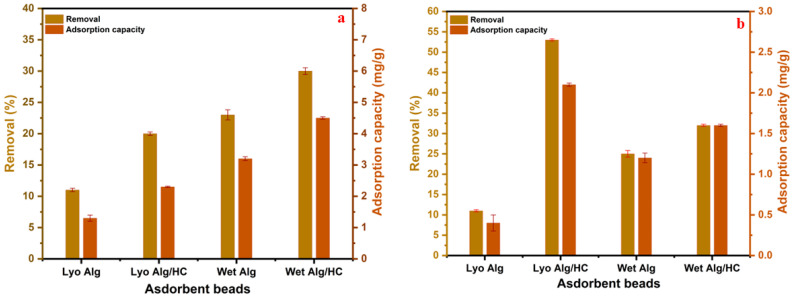
Comparison of the lyophilized and wet beads for 2-NP adsorption at 10 ppm, pH 5.2, and 60 min, with adsorbent doses of (**a**) 0.68 g_dry weight_/L and (**b**) 2 g_dry weight_/L.

Figure 5 compares the removal efficiency at both doses. At a dose of 0.68 g/L (Figure 5a), wet Alg/HC beads achieved ≈35% removal efficiency, significantly outperforming the wet Alg beads. At the higher dose of 2 g/L (Figure 5b), lyophilized Alg/HC beads reached ≈55% removal, compared to ≈10% for lyophilized Alg beads. These results highlight the critical role of hydrochar in enhancing adsorption through the introduction of additional oxygenated functional groups and increased surface heterogeneity [9].

Based on performance and practicality, the wet Alg/HC beads were selected for all subsequent experiments. They offer easier handling and eliminate the need for lyophilization, which can increase operational complexity and cost. In addition, inductively coupled plasma (ICP) analysis showed minimal copper leaching from the beads, with concentrations remaining below 0.3 mg/L after 1 h of contact time—well within environmentally acceptable limits.

#### 3.2.1. Batch Adsorption Kinetics of 2-Nitrophenol on Alginate–Hydrochar Beads

**Figure 6:**
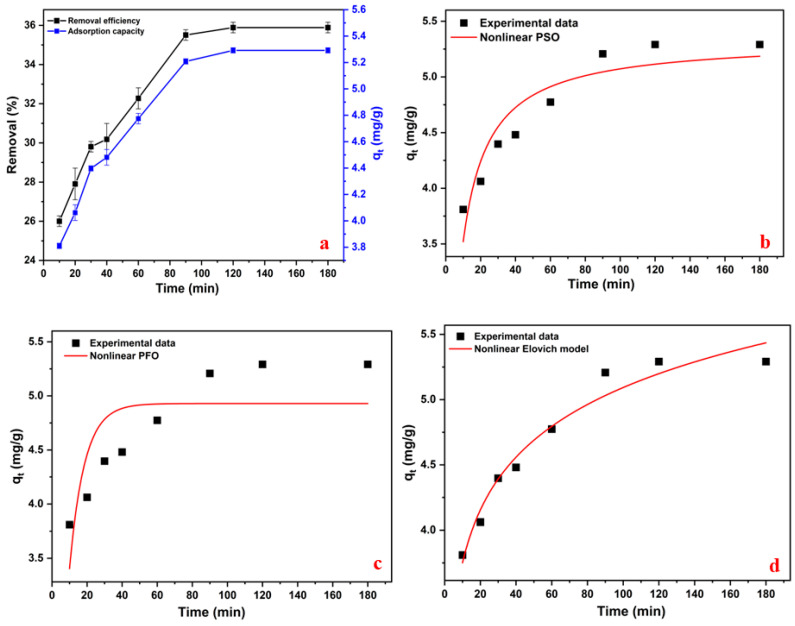
(**a**) Effect of contact time on 2-NP removal (%) and adsorption capacity (mg/g), (**b**) pseudo-second-order (PSO) kinetic, (**c**) pseudo-first-order (PFO) kinetic, and (**d**) Elovich nonlinear kinetic models.

Adsorption kinetics provide valuable insight into the mechanism and rate-limiting steps of sorption processes and are essential for modeling system design and scaling [33,34]. The effect of contact time (10–180 min) on 2-NP adsorption using wet Alg/HC beads (initial concentration: 10 mg/L) is shown in Figure 6. The adsorption rate was initially rapid due to the abundance of active sites, then gradually slowed as sites became occupied. The equilibrium was reached within 60 min, beyond which adsorption capacity remained relatively stable (Figure 6a). Compared to Isichei and Okieimen [35], who reported a maximum adsorption capacity (q_e_) of 6 mg/g at 50 mg/L, the present study achieved a q_e_ of 5.2 mg/g at only 10 mg/L, highlighting the high efficiency of Alg/HC beads at lower contaminant concentrations.

The nonlinear plots of the pseudo-second-order (PSO), pseudo-first-order (PFO), and Elovich models are shown in Figure 6b–d. Model parameters are summarized in Table S1. The PSO model provided a close match to the experimental q_e_ values and exhibited a higher R^2^ than the PFO model, confirming that the process is largely chemisorption-driven. Similar kinetic behavior has been observed for 2-NP adsorption on carbon nanotube-based adsorbents [31]. The Elovich model also fits well, suggesting surface heterogeneity and multilayer adsorption [36]. Based on AICc values (Table S1), the Elovich model provided the best fit, followed by the PSO model, while the PFO model was the least suitable.

Intraparticle diffusion was also examined to explore possible transport limitations (Table S2 and Figure S3). The adsorption profile showed two distinct phases: an initial steep slope attributed to rapid external mass transfer across the boundary layer, followed by a slower second phase corresponding to intraparticle diffusion into the hydrochar network [37]. The absence of a linear fit passing through the origin in either stage suggests that intraparticle diffusion was not the sole rate-limiting mechanism. External film diffusion and chemisorption likely acted concurrently [37].

Additionally, the effect of adsorbent mass on removal efficiency and adsorption capacity was determined (Figure S4). Removal efficiency increased with mass up to 0.7 g (0.96 g_dry/L) due to the greater availability of active sites. However, beyond 0.7 g, efficiency slightly declined, suggesting that excess adsorbent may lead to site aggregation or hinder mass transfer. Adsorption capacity continuously decreased as adsorbent mass increased, aligning with the PSO and Elovich models and indicative of chemisorption-dominated uptake on heterogeneous surfaces.

#### 3.2.2. Batch Adsorption Isotherms and Thermodynamics of 2-Nitrophenol on Alginate–Hydrochar Beads

**Figure 7:**
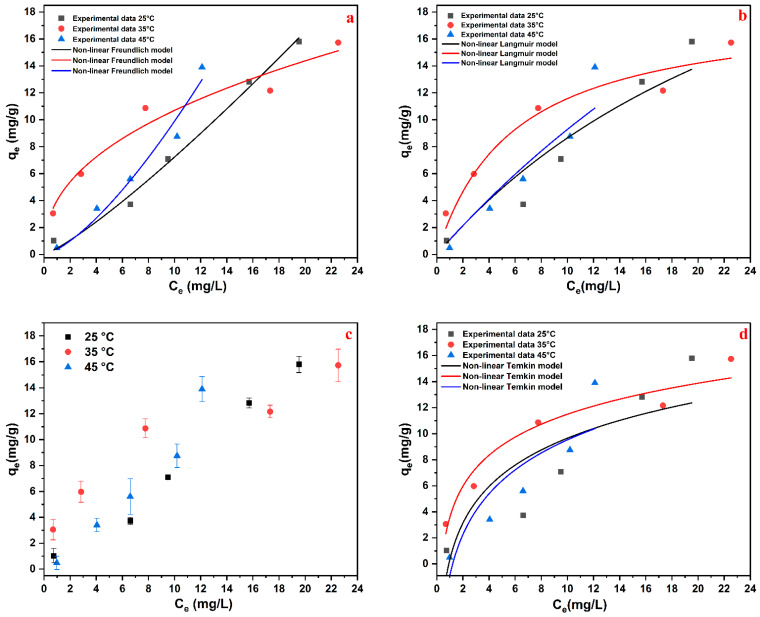
(**c**) Influence of the initial 2-NP concentration on q_e_ (mg/g); nonlinear regression plots of the (**a**) Freundlich, (**b**) Langmuir, and (**d**) Temkin models.

The isotherm was determined by exposing Alg/HC beads to 2-NP aqueous solutions at varying concentrations and temperatures, using a 1 h equilibrium time and an adsorbent dose of 0.96 g/L (dry basis). Adsorption behavior was analyzed using nonlinear Langmuir, Freundlich, and Temkin models [20,38], with details provided in the supplementary data. Table S3 contains the isotherm constants, and Figure 7 shows the nonlinear fits. Adsorption capacity increased from 1.03 mg/g to 15.80 mg/g as the initial 2-NP concentration rose from 2 ppm to 30 ppm at 25°C (Figure 7c). Higher initial concentrations improved mass transfer, enhancing the movement of 2-NP molecules to active sites [31,39]. A slight increase in adsorption capacity was observed with increasing adsorption temperature at a given initial 2-NP concentration, suggesting improved molecular diffusion and surface interaction. Abaide et al. (2019) reported an adsorption capacity of ~2 mg/g for 2-NP adsorption on hydrolyzed rice straws at 25 mg/L, 298 K, and 4 h of contact time [40]. In comparison, our study achieved 12.82 mg/g for 2-NP at 25 mg/L in just 1 h, demonstrating the superior efficiency of Alg/HC beads, particularly at low initial concentrations.

Based on the analysis of adsorption isotherm parameters, the Freundlich, Langmuir, and Temkin models exhibited different accuracies depending on temperature, with the Freundlich model providing the most consistent fit. This was confirmed by its low AICc, high R^2^, and low RMSE values, indicating heterogeneous adsorption involving both monolayer and multilayer adsorption [40]. The adsorption strength was highest at 35 °C (k_F_ = 4.01), with favorable behavior confirmed by *n* > 1 at this temperature. As shown in Figure 7b, the Langmuir model performed well at 35 °C, suggesting monolayer adsorption; however, discrepancies at 25 °C and 45 °C indicated limitations in representing the experimental data. Figure 7d shows that the Temkin model moderately fitted the data at 35 °C but poorly described the data at 25 °C and 45 °C, likely due to its assumption of uniform adsorption energy, which may not hold for the heterogeneous Alg/HC surface. Overall, the Freundlich model was the most reliable across all temperatures, reflecting heterogeneous adsorption behavior. These findings align with kinetic results, where the Elovich and pseudo-second-order models confirmed chemisorption-dominated adsorption on active sites with varying energy. Furthermore, the sequential occupation of active sites—starting from external surface adsorption to internal pore diffusion—supports the Freundlich isotherm’s multilayer adsorption mechanism.

Thermodynamic parameters provide comprehensive insight into the energetic changes associated with adsorption [41]. The standard Gibbs energy change (ΔG°), enthalpy change (ΔH°), and entropy change (ΔS°) are described in the supplementary data. The calculated thermodynamic parameters are provided in Table 1, with supporting details provided in the supplementary data. At an initial 2-NP concentration of 10 mg/L, ΔH° was positive between 298 and 308 K, indicating an endothermic adsorption process. However, at 318–328 K, the process became exothermic, suggesting that adsorption weakened due to reduced adsorbate–adsorbent interactions at high temperatures. This temperature-dependent behavior is not indicative of a transition from physisorption to chemisorption but reflects the effect of surface heterogeneity and variable adsorption site energies, which are characteristic of biopolymer-based porous materials [41]. These results align with the Freundlich and Elovich models, which describe adsorption on a heterogeneous surface with mixed thermodynamic behavior. The positive ΔS° at 298–308 K suggested increased molecular disorder and strong 2-NP affinity for the composite beads, likely due to adsorbate restructuring at the solid–solution interface. At a higher 2-NP concentration (30 mg/L), the adsorption process was exothermic, indicating preferential adsorption on low-energy sites or site saturation, where fewer high-energy sites remained available for adsorption. In general, the observed thermodynamic variations are coherent with a physical adsorption mechanism driven by multiple interaction types and site-specific energies. This interpretation is further supported by the stability of desorption performance observed over four cycles, as discussed in the section on Packed-Bed Adsorbent Bead Regeneration.

### 3.3. Fixed-Bed Adsorption Performance

**Figure 8:**
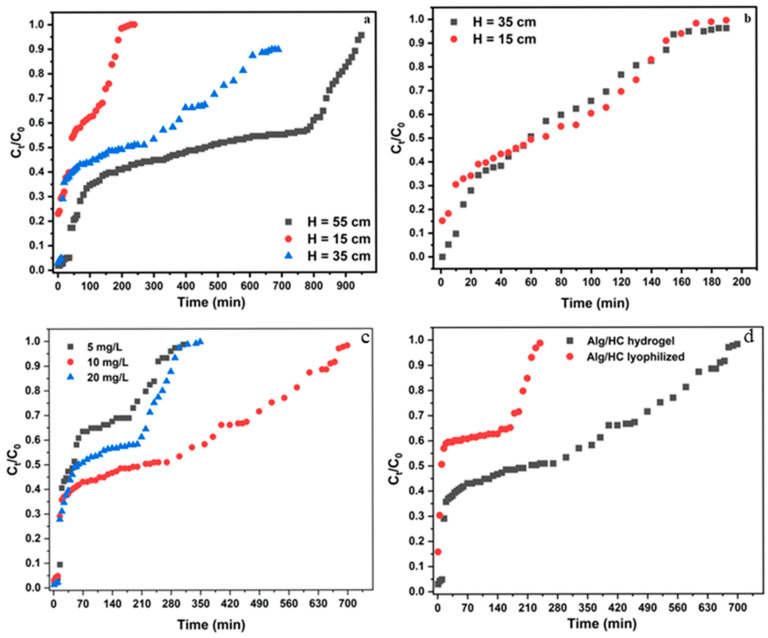
Breakthrough curves for 2-NP adsorption: (**a**) effect of bed height at 3.8 mL/min, (**b**) effect of bed height at 5.2 mL/min, (**c**) effect of initial 2-NP concentration, and (**d**) comparison of lyophilized vs. hydrogel beads (3.8 mL/min, 10 mg/L).

The inflow rate is a critical parameter, as it regulates the contact time between the solute and adsorbent surface. The continuous adsorption of 2-NP by the Alg/HC hydrogel beads was studied using two flow rates, with constant bed heights of 15 cm and 35 cm and an initial 2-NP concentration of 10 mg/L (Figure S6). At the higher flow rate (5.2 mL/min), breakthrough occurred more quickly, meaning the effluent 2-NP concentration reached 10% of the influent concentration in a shorter time due to reduced contact time between the adsorbate and adsorbent. In contrast, the lower flow rate (3.8 mL/min) extended breakthrough time, improving adsorption efficiency. Similarly, increasing the bed height to 35 cm significantly improved adsorption efficiency, as it provided more active sites and prolonged contact time, leading to a delayed breakthrough and extended saturation time compared to the 15 cm bed height. Figure 8 illustrates the effect of bed height on breakthrough curves at two flow rates. As shown in Figure 8a, at a fixed flow rate of 3.8 mL/min, the breakthrough time (t_b_) occurred at 0, 12, and 35 min for bed heights of 15, 35, and 46 cm, respectively, while the saturation time (t_s_) extended from 180 to 930 min as bed height increased. These findings indicate that a longer bed depth increased the mass transfer zone, extending the adsorption region and enhancing contact time between 2-NP molecules and active sites. Similar trends have been reported by Mahmoud et al. (2022) and Zheng et al. (2024) [12,42].

The influence of 2-NP concentration on adsorption capacity is a crucial parameter because it determines the maximum amount of 2-NP that can be removed by a given mass of adsorbent. To assess the effect of concentration on the breakthrough curves, fixed-bed column adsorption studies were conducted at three different concentrations, as shown in Figure 8c. Continuous 2-NP adsorption was more efficient at lower concentrations, as slower adsorbent saturation allowed for extended contaminant removal. To further explore adsorption potential under different conditions, lyophilized beads were tested in a continuous adsorption system (Figure 8d). While hydrogel beads exhibited superior performance, lyophilized beads remained a viable alternative in situations where wet beads are impractical. Both adsorbent form (hydrogel vs. lyophilized) and initial 2-NP concentration played a critical role in determining adsorption efficiency and column longevity. The breakthrough occurred faster at higher concentrations (20 mg/L) due to increased competition for the binding sites, leading to quicker saturation of the adsorbent—as indicated by the steeper slope in Figure 8c. The similar shape of breakthrough curves at 5 mg/L and 20 mg/L suggests that adsorption was influenced by a common rate-controlling step, likely mass transfer limitations (external film diffusion or intraparticle diffusion), as demonstrated in the 2-NP batch adsorption isotherm study. At 10 mg/L, adsorption exhibited intermediate behavior, indicating a mass transfer-controlled process. This trend aligns with batch adsorption findings, where the data fit well to the pseudo-second-order and Elovich models. The parameters of the Alg/HC hydrogel bead fixed-bed column for the 2-NP study, obtained from the breakthrough curves, are provided in Table S4 (supplementary data).

### 3.4. Modeling of the Breakthrough Curves

The dynamic adsorption behavior of 2-NP on Alg/HC hydrogel beads in a fixed-bed system was evaluated using four common models: Thomas, Bohart–Adams, Yoon–Nelson, and Clark. The Thomas model assumes Langmuir isotherm behavior and pseudo-second-order kinetics and is mainly suitable for systems where external and internal diffusion are not rate-limiting [43]. The Yoon–Nelson model predicts breakthrough time based on the assumption that the rate decreases proportionally to the probability of adsorbate breakthrough [11], while the Bohart–Adams model is only applicable to the initial portion of the breakthrough curve [44]. The Clark model includes the limitations of mass transfer and assumes a Freundlich-type isotherm, making it suitable for heterogeneous adsorbents [45].

Among the models, the Thomas model, based on pseudo-second-order kinetics, best described adsorption as a chemisorption-driven process rather than simple diffusion. As shown in Table 2, the Thomas model exhibited strong predictive accuracy (R^2^ = 0.961, AICc = −155 at Z = 15 cm), particularly at lower flow rates and greater bed heights, reinforcing batch study findings that adsorption occurred through strong adsorbate–adsorbent interactions [21]. The Clark model also demonstrated robust performance (R^2^ = 0.969, AIC_c_ = −162 at Z = 35 cm and Q = 5.2 mL/min), supporting the presence of heterogeneous adsorption sites, aligning well with batch-scale Freundlich isotherm results [45].

The Bohart–Adams model adequately described the early stages of adsorption, particularly at lower flow rates (R^2^ = 0.963, AICc = −15 at Z = 35 cm, Q = 3.8 mL/min), suggesting the contribution of external mass transfer. However, the model’s performance decreased at higher bed heights (R^2^ = 0.886 at Z = 55 cm), possibly due to intraparticle diffusion effects, which are not considered by the model. The Yoon–Nelson model provided consistent predictions for all bed heights and concentrations, reinforcing its usefulness for simplified estimation of breakthrough times under plug flow conditions [46]. Overall, these results would suggest that 2-NP adsorption in the column system is governed by a combination of film diffusion, site heterogeneity, and favorable kinetics, with the Thomas and Clark models offering the best predictive accuracy for system design.

### 3.5. Packed-Bed Adsorbent Bead Regeneration

**Figure 9:**
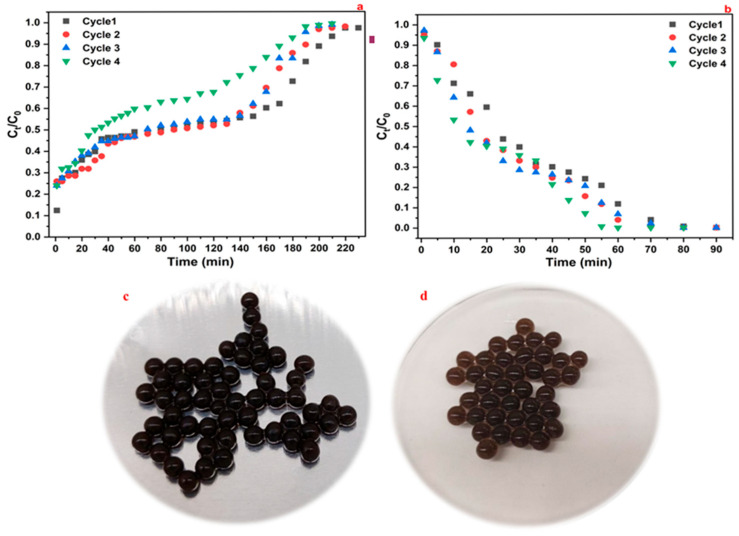
(**a**) Breakthrough curves for the 2-NP removal cycles, (**b**) desorption curves for the 2-NP elution cycles, (**c**) adsorbent beads before regeneration, and (**d**) adsorbent beads after regeneration.

Adsorbent regeneration is a critical step to enable reuse across multiple cycles, enhancing the cost-effectiveness of the process [15,43]. Therefore, the regeneration and reusability properties of the Alg/HC hydrogel beads in a continuous system were investigated. Figure 9 illustrates the regeneration study of the Alg/HC hydrogel beads. The breakthrough curves had a progressive shift in Cycle 4 (Figure 9a), where breakthrough occurred earlier, indicating a decrease in adsorption capacity due to adsorbent surface saturation. However, the desorption curves (Figure 9b) demonstrated consistent elution behavior, with complete desorption achieved within 60 min across all cycles. As shown in Table S5, the eluted amount of 2-NP initially increased from 3.23 mg/g (Cycle 1) to 4.10 mg/g (Cycle 3), suggesting improved desorption efficiency due to pore expansion or enhanced adsorption kinetics. However, a decrease to 3.17 mg/g (Cycle 4) suggested potential adsorbent fatigue.

The desorption efficiency (%El) increased steadily, reaching 90% in cycle 4. This suggests that while adsorption efficiency decreased slightly, desorption remained highly efficient, which suggests that the composite beads maintain their functional integrity and are suited to multiple cycles of reuse. The ethanol–water mixture used as eluent proved both effective and environmentally friendly, enhancing elution performance without damaging the beads’ structure. A visual inspection (Figure 9c,d) confirmed that the beads retained their shape and surface pattern after regeneration, confirming their physical stability.

## 4. Conclusions

This study investigated the feasibility of using HC derived from waste-contaminated biomass in composite hydrogel beads for 2-NP removal in a batch and fixed-bed adsorption systems. Batch adsorption experiments confirmed that the process followed a heterogeneous adsorption mechanism, as indicated by the strong fit to the pseudo-second-order, Elovich, and Freundlich models. Fixed-bed column experiments demonstrated that adsorption efficiency improved at lower flow rates and higher bed heights, enhancing contact time and adsorbent site utilization. Among the evaluated models, the Yoon–Nelson model most accurately predicted breakthrough behavior, while the Bohart–Adams model effectively described only the initial adsorption phase. Additionally, hydrogel bead regeneration was successfully assessed over four consecutive adsorption–desorption cycles, confirming high desorption efficiency and reusability of the Alg/HC beads. These findings establish hydrochar-based hydrogel beads as a promising and reusable adsorbent for sustainable wastewater treatment applications. This study initiates a pathway for future research concentrating on more detailed insight into the adsorption mechanisms, long-term performance, and scale-up feasibility to confirm its suitability for industrial use.

## Figures and Tables

**Table 1:** Thermodynamic parameters for the adsorption of 2-NP onto the Alg/HC beads.

2-NP Concentration (mg/L)	Temperature (K)	ΔG° (kJ/mol)	ΔH° (kJ/mol)	ΔS° (J/mol K)
**10**	298.15	1.42	100.69	332.94
	308.15	−1.90		
	318.15	0.46	−41.50	−131.88
	328.15	1.78		
**30**	298.15	0.52	−11.30	−39.64
	308.15	0.92		
	318.15	−0.36	−47.14	−147.03
	328.15	1.10		

**Table 2:** Nonlinear fitting parameters of the dynamic models for the 2-NP adsorption breakthrough curves.

**Column adsorption experiments**	Q (mL/min)	3.8	3.8	3.8	5.2	3.8	3.8
	Z (cm)	35	55	15	35	35	35
	C_0_ (mg/L)	10	10	10	10	20	5
**Thomas model**	K_Th_ (mL/(mg min))	0.0004	0.0003	0.002	0.003	0.0005	0.003
	q_0_ (mg/g)	235	337	152	97	235	40
	R^2^	0.867	0.803	0.946	0.961	0.838	0.817
	RMSE	0.07	0.008	0.05	0.06	0.10	0.11
	AIC_C_	−264	−484	−174	−155	−184	−158
**Yoon–Nelson model**	K_YN_ (min^−1^)	0.004	0.003	0.016	0.03	0.01	0.012
	τ (min)	212	488	60	65	108	73
	R^2^	0.872	0.803	0.946	0.956	0.838	0.817
	RMSE	0.01	0.08	0.05	0.06	0.10	0.11
	AIC_C_	−158	−482	−172	−155	−183	−158
**Bohart–Adams model**	K_AB_ (mL/(mg min))	0.030	0.003	0.002	0.010	0.006	0.024
	N_0_ (mg/L)	21	72	176	46	61	19
	R^2^	0.963	0.886	0.915	0.924	0.815	0.934
	RMSE	0.020	0.030	0.000	0.030	0.060	0.000
	AIC_C_	−15	−92	−21	−25	−7	−23
**Clark model**	A	0.019	0.012	1.662	0.018	−1.0	0.007
	*n*	1.0	1.0	1.7	1.0	−0.9	1.0
	r (min^−1^)	0.003	0.002	0.015	0.020	0.004	0.010
	R^2^	0.868	0.814	0.941	0.969	0.873	0.840
	RMSE	0.075	0.085	0.057	0.049	0.089	0.101
	AIC_C_	−262	−486	−169	−162	−193	−164

## Data Availability

The original contributions presented in this study are included in the article/Supplementary Material. Further inquiries can be directed to the corresponding author.

## Supplementary Materials

The following supporting information can be downloaded at: https://www.mdpi.com/article/10.3390/ma18102412/s1, Figure S1: Experimental setup for the preparation of Alg/HC hydrogel beads; Figure S2: (a) Digital image of the Alg/HC hydrogel beads and (b) size distribution (average of 60 beads); Table S1: Kinetic parameters for 2-NP adsorption onto the Alg/HC hydrogel beads; Figure S3: Intra-particle diffusion kinetic model; Table S2: Intra-particle diffusion kinetic parameters for the removal of 2-NP onto Alg/HC beads; Figure S4: Effect of adsorbent dose for the removal of 2-NP (concentration of 2-NP, 10 mg/L; ambient temperature; contact time, 60 min); Table S3: Equilibrium isotherm models at various temperatures for 2-NP adsorption onto the Alg/HC beads; Figure S5: Van’t Hoff plots using piecewise regression analysis of (a) 10 mg/L and (b) 30 mg/L initial 2-NP concentrations; Figure S6: Effect of flow rate on the breakthrough curves for 2-NP adsorption at different bed heights: (a) 15 cm and (b) 35 cm; Table S4: Parameters of the Alg/HC hydrogel bead fixed-bed column for the 2-NP study obtained from the breakthrough curves; Table S5: Desorption cycle efficiency parameters.
