# Supplementary material for: Sustainable Alginate–Hydrochar Composite Beads for 2-Nitrophenol Adsorption in Batch and Fixed-Bed Systems
Source: Materials (Basel). 2025 May 21;18(10):2412. doi: 10.3390/ma18102412 (PMC12112803; doi:10.3390/ma18102412)
Supplement: Supplementary file 1 [file materials-18-02412-s001.zip › materials-3618654-supplementary.pdf]

Sustainable Alginate–Hydrochar Composite Beads for 2-Nitrophenol Adsorption in Batch and Fixed-Bed Systems

Dalia Allouss[1], Nicolas Abatzoglou[1], and Inès Esma Achouri*[1]

[1]Group of Research on Technologies and Processes, Department of Chemical and Biotechnological Engineering, Université de Sherbrooke, Sherbrooke, QC J1K 2R1, Canada

E-mail: ines.esma.achouri@usherbrooke.ca (*Corresponding Author)

Materials and methods

Alginate-hydrochar composite bead preparation

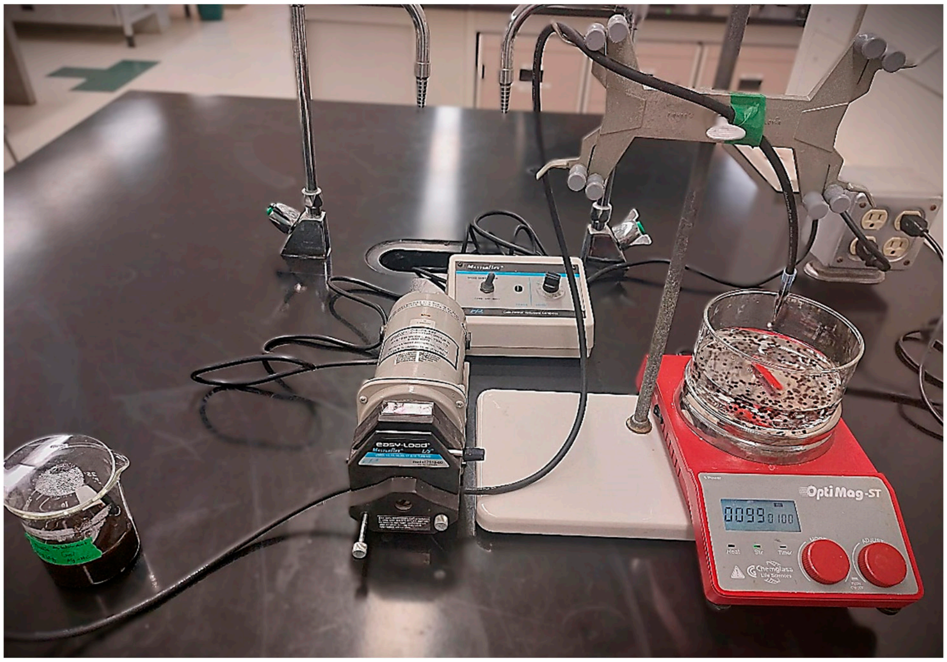

Figure S1. Experimental setup for the preparation of Alg/HC hydrogel beads.

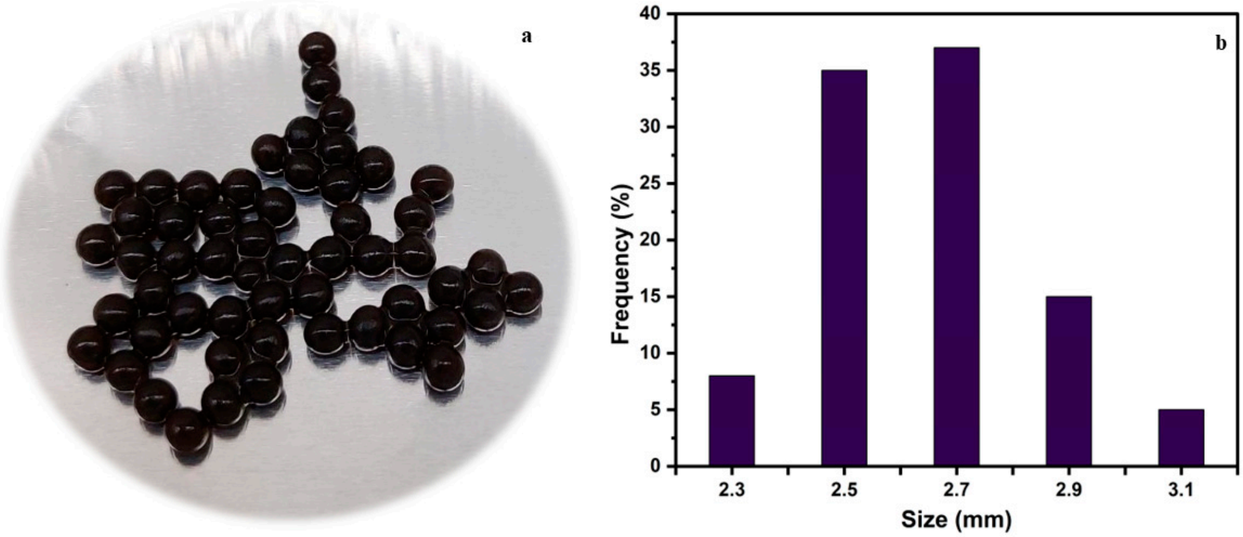

Figure S2 (a) Digital image of the Alg/HC hydrogel beads and (b) size distribution (average of 60 beads).

2-Nitrophenol batch adsorption experiments

$$q_e = \frac{C_0 - C_e}{m} \times V \qquad \textit{Eq.S 1}$$

$$R = \frac{C_0 - C_e}{C_0} \times 100 \qquad \textit{Eq.S 2}$$

Where C_0 (mg/L) is the initial 2-NP concentration, C_e (mg/L) is the equilibrium concentration, m (g) is the adsorbent mass, and V (L) is the solution volume.

Kinetic modeling

Kinetic models, including pseudo-first order, pseudo-second order, Elovich and intraparticle diffusion, were applied to analyze the adsorption rate and rate-controlling steps on batch study data. The nonlinear forms of these models are presented below, respectively.

$$q_t = q_{e,cal}\,(1 - e^{-k_1 t}) \qquad \textbf{Eq.S 3}$$

$$q_t = \frac{k_2\, q_{e,cal}\, t}{1 + k_2\, q_{e,cal}\, t} \qquad \textbf{Eq.S 4}$$

$$q_t = \frac{ln\,(\alpha\beta t)}{\beta} \qquad \textbf{Eq.S 5}$$

$$q_t = k_i\, t^{1/2} + C \qquad \textbf{Eq.S 6}$$

Where k_1 is the kinetic constant of pseudo-first order kinetics (min^{-1}), while k_2 is the rate constant for pseudo-second-order kinetics (g/mg.min). The adsorption capacities at equilibrium and at time t are denoted by q_e (mg/g) and q_t (mg/g), respectively.

Modeling of the adsorption isotherms and thermodynamics

The batch adsorption behavior was investigated using Freundlich, Langmuir, and Temkin models, and the non-linearized forms are expressed as the following equations [1].

Freundlich model :

$$q_e = k_f\, C_e^{1/n} \qquad \textbf{Eq.S 7}$$

Langmuir model :

$$q_e = \frac{q_m k_L C_e}{1 + k_L C_0} \qquad \textbf{Eq.S 8}$$

$$R_L = \frac{1}{1 + k_L C_0} \qquad \textbf{Eq.S 9}$$

Temkin model :

$$q_e = \frac{RT}{B}\, ln\,(A_T C_e) \qquad \textbf{Eq.S 10}$$

Where q_e is the adsorbed solute (mg/g), C_e is the equilibrium concentration (mg/L), k_L and R_L are Langmuir coefficient and dimensionless equilibrium parameter, respectively. k_f and n are Freundlich coefficient and heterogeneity factor, respectively. R (J/mol K) represents the universal gas constant, T (K) denotes the temperature, B (L/g) is the Temkin constant associated with the heat of adsorption, and A_T is the Temkin constant related to the adsorption potential.

$$K_L = \frac{q_e}{C_e} \qquad \textbf{Eq.S 11}$$

$$\Delta G^\circ = -RT lnK_L \qquad \textbf{Eq.S 12}$$

$$\Delta G^\circ = \Delta H - T\Delta S^\circ \qquad \textbf{Eq.S 13}$$

$$lnK = \frac{\Delta S^\circ}{R} - \frac{\Delta H^\circ}{RT} \qquad \textbf{Eq.S 14}$$

Where K_L represents the distribution coefficient, T is the temperature, and R denotes the gas constant with a value of 8.3145 J/mol K.

Batch adsorption performance

Batch adsorption kinetics of 2-Nitrophenol on alginate–hydrochar beads

Table S1 Kinetic parameters for 2-NP adsorption onto the Alg/HC hydrogel beads.

Model	Parameter
**Pseudo-first-order kinetic**	k_1 = 0.12 min^{-1}<br>$q_{e,calc}$ = 4.93 mg/g<br>$q_{e,exp}$ = 5.29 mg/g<br>R^2 = 0.566<br>R^2_{adj} = 0.493<br>RMSE = 0.35<br>AIC_c = −10.27
**Pseudo-second-order kinetic**	k_2 = 0.036 g/mg.min<br>$q_{e,calc}$ = 5.33 mg/g<br>$q_{e,calc}$ = 5.29 mg/g<br>R^2 = 0.874<br>R^2_{adj} = 0.853<br>RMSE = 0.19<br>AIC_c = −20.20
**Elovich**	α = 36.26 mg/g.min<br>β = 1.72 g/mg<br>RMSE = 0.09<br>R^2 = 0.965<br>R^2_{adj} = 0.959<br>AIC_c = −32.54

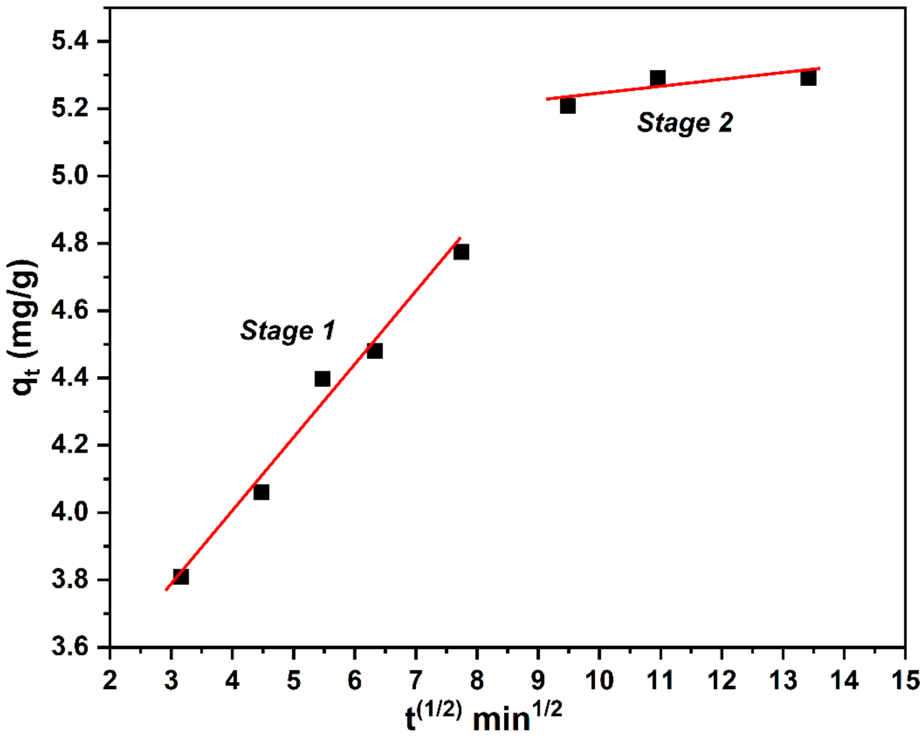

Figure S3. Intra-particle diffusion kinetic model.

Table S2. Intra-particle diffusion kinetic parameters for the removal of 2-NP onto Alg/HC beads.

Parameters	Stage (1)	Stage (2)
**k_i (mg/g min$^{1/2}$)**	0.21	0.02
**C_i (mg/g)**	3.15	5.05
**R^2**	0.98	0.62
**RMSE**	0.04	0.02

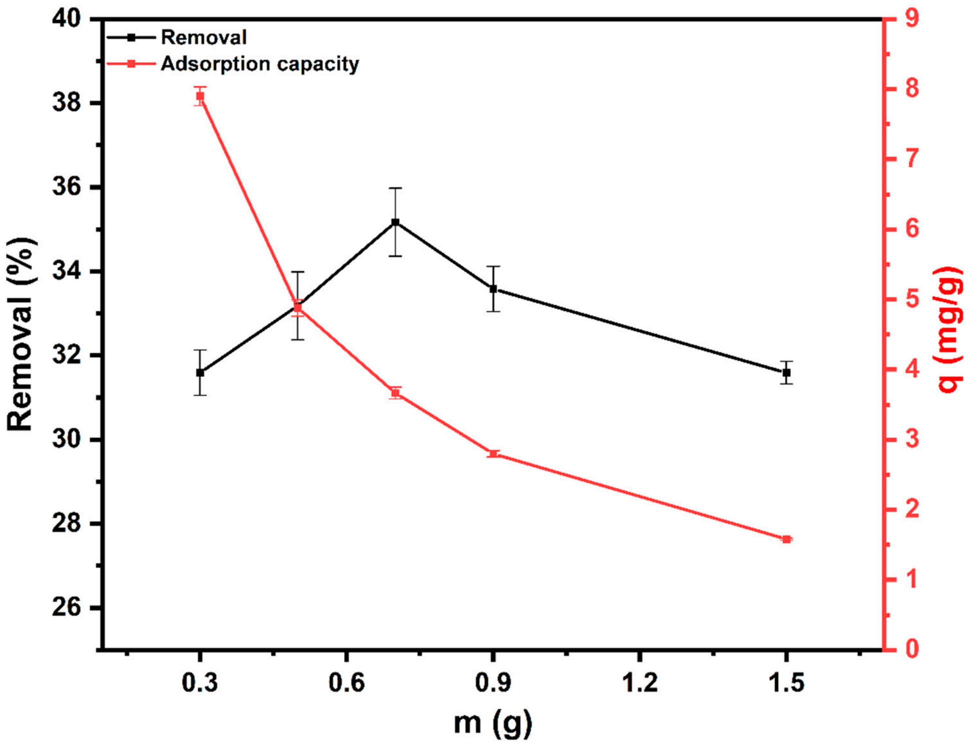

Figure S4. Effect of adsorbent dose for the removal of 2-NP (concentration of 2-NP, 10 mg/L; ambient temperature; contact time, 60 min).

Batch adsorption isotherms and thermodynamics of 2-Nitrophenol on alginate–hydrochar beads

Table S3 Equilibrium isotherm models at various temperatures for 2-NP adsorption onto the Alg/HC beads.

Nonlinear isotherm model	Isotherm parameter	Alg/HC hydrogel beads		
		25 °C	35 °C	45 °C
Freundlich	k_F	0.46	4.01	0.38
	n	0.84	2.34	0.70
	R^2	0.991	0.960	0.968
	R^2_{adj}	0.988	0.947	0.958
	RMSE	0.51	0.89	0.81
	AIC_c	3.25	8.91	3.95
Langmuir	q_m (mg/g)$_{experimental}$	15.80	15.73	13.90
	q_m (mg/g)$_{estimated}$	36.08	18.37	57.50
	k_L (L/mg)	0.03	0.17	0.02
	R_L	0.5–0.9	0.1–0.7	0.6–0.9
	R^2	0.915	0.991	0.893
	R^2_{adj}	0.887	0.988	0.857
	RMSE	1.60	0.88	1.50
	AIC_c	14.68	6.82	12.10
Temkin	A_T	1.08	2.83	0.82
	B	0.25	0.30	0.22
	R^2	0.732	0.945	0.779
	R^2_{adj}	0.643	0.927	0.706
	RMSE	2.85	1.05	2.17
	AIC_c	16.48	6.54	13.73

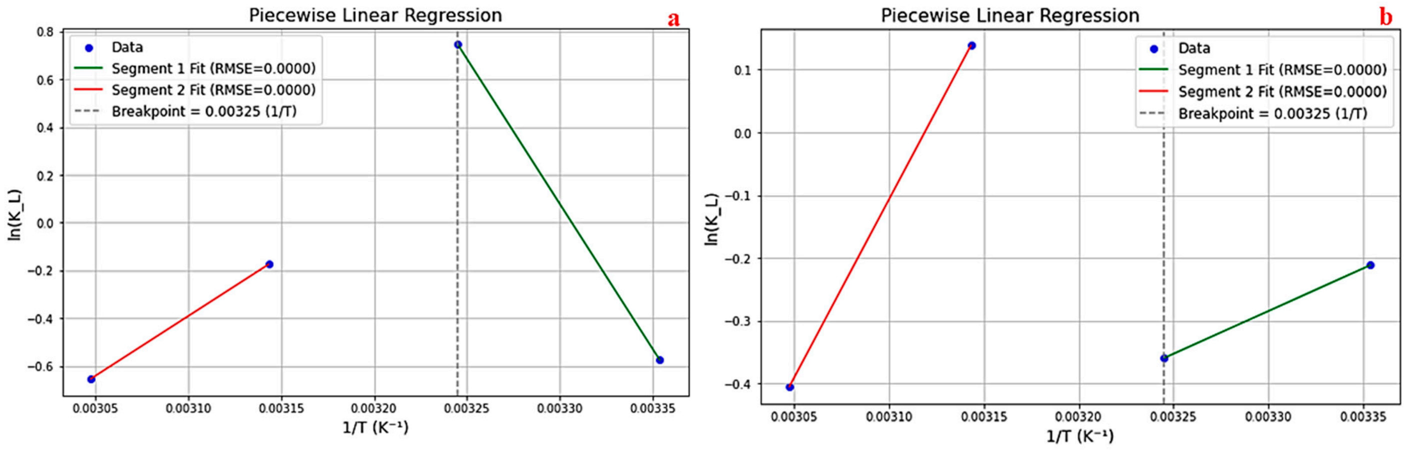

Figure S5. Van't Hoff plots using piecewise regression analysis of (a) 10 mg/L and (b) 30 mg/L initial 2-NP concentrations.

Fixed-bed adsorption performance

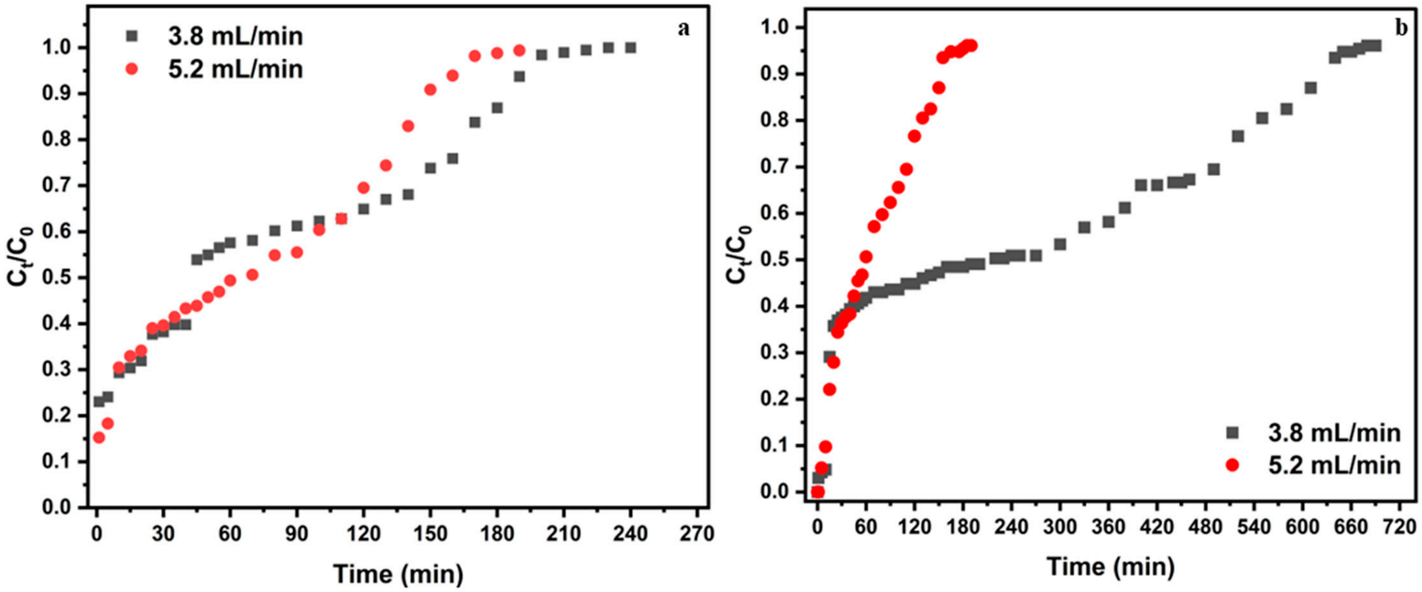

Figure S6 Effect of flow rate on the breakthrough curves for 2-NP adsorption at different bed heights: (a) 15 cm and (b) 35 cm.

Table S4 Parameters of the Alg/HC hydrogel bead fixed-bed column for the 2-NP study obtained from the breakthrough curves.

Conditions	t_b (min)	t_s (min)	Δt (min)	Z_m (cm)	V_{eff} (mL)	q_{total} (mg)	q_e (mmol/g)	m_{total} (mg)	%R
**C_0 = 20 mg/L H = 35 cm Q = 3.8 mL/min**	12	284	272	34	1,330	8.5	33.8	26.6	32
**C_0 = 10 mg/L H = 35 cm Q = 3.8 mL/min**	11	656	645	34	2,660	9.8	38.7	26.6	37
**C_0 = 5 mg/L H = 35 cm Q = 3.8 mL/min**	15	247	232	33	1,178	1.5	6.1	6.0	26
**C_0 = 10 mg/L H = 35 cm Q = 5.2 mL/min**	10	180	170	33	988	3.8	15.3	9.8	39
**C_0 = 10 mg/L H = 15 cm Q = 3.8 mL/min**	0	180	180	15	912	3.2	26.4	9.1	35
**C_0 = 10 mg/L H = 35 cm Q = 3.8 mL/min**	12	623	611	34	2,622	9.9	39.0	26.2	38
**C_0 = 10 mg/L H = 46 cm Q = 3.8 mL/min**	35	930	895	44	3,610	16.3	49.1	36.10	45

Modeling of the breakthrough curves

Thomas model

$$\frac{C}{C_0} = \frac{1}{1 + exp\,(\frac{k_{TH} q_0 m}{Q} - K_{TH} C_0 t)}$$

Bohart-Adams model

$$\frac{C}{C_0} = exp\,(K_{BA} C_0 t - K_{BA} N_0 \frac{Z}{Q})$$

Yoon-Nelson model

$$\frac{C}{C_0} = \frac{exp\,(K_{YN} t - K_{YN} \tau)}{1 + exp\,(K_{YN} t - K_{YN} \tau)}$$

Clark model

$$\frac{C}{C_0} = (\frac{1}{1 + Ae^{-rt}})^{\frac{1}{n-1}}$$

Where K_{BA}k (L/mg·min) is the Bohart-Adams constant, N_0 (mg/L) is the saturation concentration, Z (cm) is the bed depth of the column, and Q (Ml/min) is the inflow rate. The parameter k_{YN} (min−1) represents the Yoon-Nelson kinetic constant, while τ (min) is the contact time required for 50% breakthrough of the adsorbate. Additionally, k_{TH} (Ml/mg·min) is the Thomas kinetic constant. The parameter A is a constant related to the initial conditions, and r is the rate constant that determines the rate at which the micropollutant concentration changes over time, and n is the Hill coefficient.

Packed-bed adsorbent beads regeneration

$$q_{el} = \frac{Q}{1000 \times m} \int_{t=0}^{t=t_{total}} C_{el}\, dt$$

$$\%El = \frac{q_{el}}{q_e} \times 100$$

Where, C_{el} is the concentration of the eliminated 2-NP over time.

Table S5 Desorption cycle efficiency parameters.

Parameter	Cycle 1	Cycle 2	Cycle 3	Cycle 4
**q_{el} (mg/g)**	3.23	3.57	4.10	3.17
**%El (%)**	64	73	77	90

References

1. Khadhri, N.; El Khames Saad, M.; Ben Mosbah, M.; Moussaoui, Y. Batch and Continuous Column Adsorption of Indigo Carmine onto Activated Carbon Derived from Date Palm Petiole. *J Environ Chem Eng* **2019**, *7*, 102775, doi:10.1016/j.jece.2018.11.020.
